# Radiomics features from the peritumoral region can be associated with the epilepsy status of glioblastoma patients

**DOI:** 10.3389/fonc.2025.1587745

**Published:** 2025-08-25

**Authors:** Yeong Chul Yun, Johann M. E. Jende, Katharina Holz, Sabine Wolf, Freya Garhöfer, Anja Hohmann, Philipp Vollmuth, Martin Bendszus, Heinz-Peter Schlemmer, Felix Sahm, Sabine Heiland, Wolfgang Wick, Varun Venkataramani, Felix T. Kurz

**Affiliations:** ^1^ Department of Neuroradiology, Heidelberg University Hospital, Heidelberg, Germany; ^2^ Division of Radiology, German Cancer Research Center, Heidelberg, Germany; ^3^ Faculty of Medicine, Heidelberg University, Heidelberg, Germany; ^4^ Department of Neurology, Heidelberg University Hospital, Heidelberg, Germany; ^5^ Department of Neuropathology, Heidelberg University Hospital, Heidelberg, Germany; ^6^ Clinical Cooperation Unit Neuropathology, German Cancer Consortium (DKTK), German Cancer Research Center (DKFZ), Heidelberg, Germany; ^7^ Department of Functional Neuroanatomy, Heidelberg University, Heidelberg, Germany; ^8^ Department of Neuroradiology, Geneva University Hospitals, Geneva, Switzerland

**Keywords:** glioblastoma, epilepsy, radiomics, MRI, machine learning, radiomics features from peritumoral

## Abstract

**Purpose:**

Identifying radiomics features that help predict whether glioblastoma patients are prone to developing epilepsy may contribute to an improvement of preventive treatment and a better understanding of the underlying pathophysiology.

**Materials and methods:**

In this retrospective study, 3-T MRI data of 451 pretreatment glioblastoma patients (mean age: 61.2 ± 11.8 years; 268 men, 183 women) were analyzed. Three hundred thirty-six patients reported no epilepsy, while 115 patients were diagnosed with symptomatic epilepsy. A total of 1,546 radiomics features were extracted from contrast-enhancing tumor, peritumoral regions, and normal-appearing white matter as regions of interest using PyRadiomics. The dataset was initially split into a training (70%) and a validation (30%) cohort. The training cohort was used for feature selection with ElasticNet and model optimization. Various machine learning models, including logistic regression (LR), were used to predict epilepsy status. The models’ performances were evaluated with the validation cohort, and the area under the curve of the receiver operating characteristics (AUC) was used as a measure. For identifying relevant features, permutation feature importance was applied.

**Results:**

The performance of LR using radiomics features from only a single ROI in the validation cohort was AUC = 0.83 (95% CI: 0.76–0.91) and AUC = 0.77 (95% CI: 0.69–0.85) for the peritumoral and white matter regions, respectively. The most important features in peritumoral regions were shape features, while for the white matter region, higher-order features from FLAIR were most relevant.

**Conclusion:**

Radiomics features from peritumoral and normal-appearing white matter can be associated with epilepsy status at diagnosis, suggesting an important role of these regions for the development of epilepsy in glioblastoma patients.

## Introduction

Glioblastoma is one of the most common primary tumors of the central nervous system (1). A variety of neurological symptoms at diagnosis is associated with this tumor, including, for example, headaches, deficits in sensomotoric functions, loss of cognitive functions, and changes in personality (2). For 30%–50% of glioblastoma patients, the tumor is associated with epilepsy (3). Regardless of the etiology of epilepsy, this neurological condition can severely compromise patients’ quality of life.

Radiomics is a method to extract quantitative features from diagnostic radiologic images in a high-throughput manner (4, 5). It can contribute to a more objective evaluation of radiological data. The strength of radiomics in a neuro-oncology setting has been demonstrated in several previous studies to identify genetic mutations of gliomas or for glioma grading (6–8).

Identifying MRI-based radiomics features that are associated with epilepsy status for glioblastoma patients can contribute to a better understanding of epilepsy in the context of neuro-oncology. Multiple other works have shown the important role of the peritumoral region for developing epileptic seizures (9, 10). This finding is supported by multiple other studies where changes in molecular and cellular environments in both tumor and peritumoral regions have been associated with epileptic seizures (11, 12). To assess whether changes to the normal-appearing white matter region (WM) are also of importance for the development of epilepsy, the region of interest (ROI) for radiomics feature extraction in our study was not limited to the contrast-enhancing tumor region (CET) and the non-enhancing lesion (NEL) presenting as a hyperintense lesion in T2w/FLAIR images. Instead, radiomics features extracted from the peritumoral region surrounding the CET (PeriCET) and WM were included as well.

## Materials and methods

### Patients

**Figure 1:**
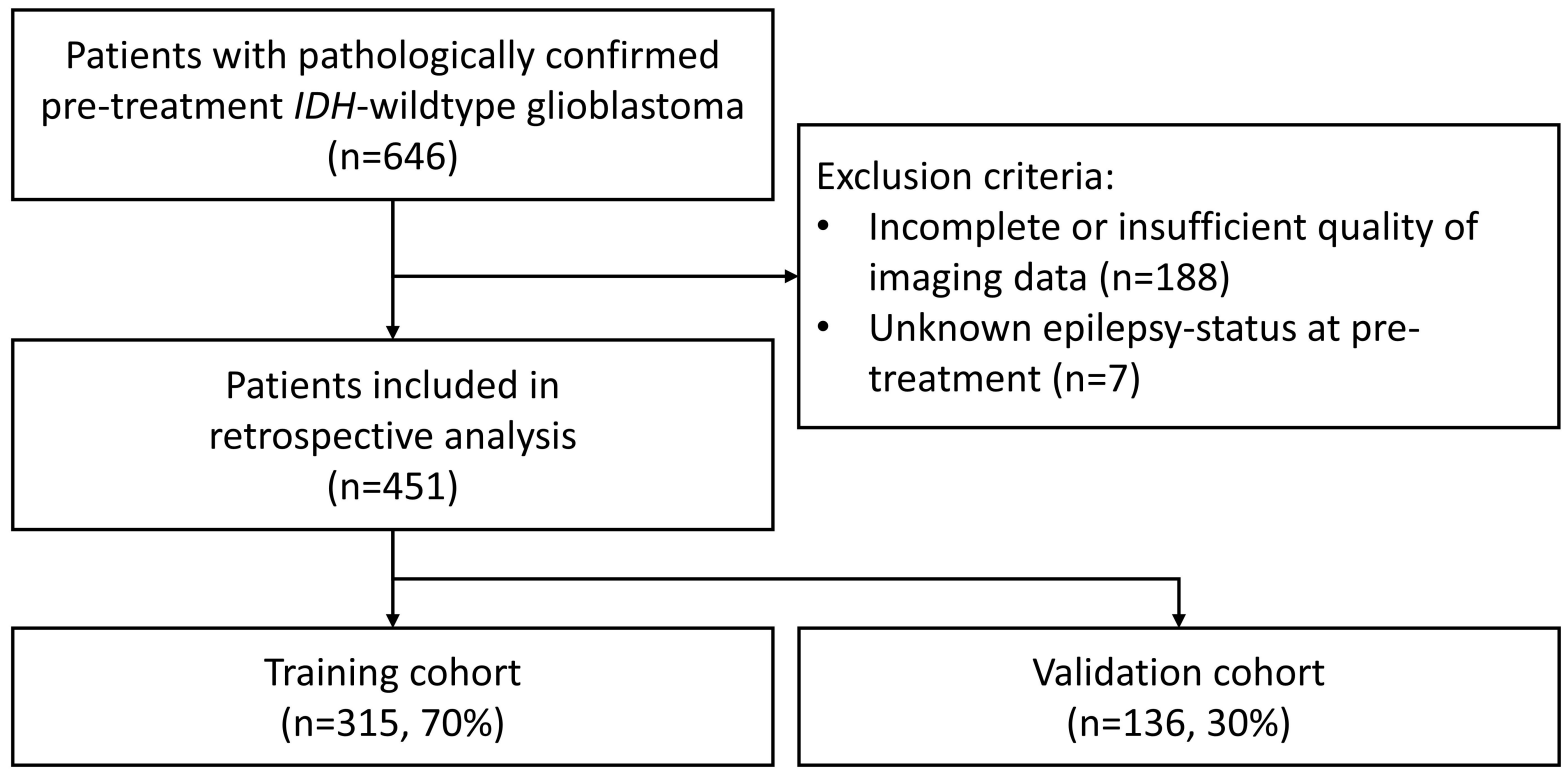
Flow diagram of the study population showing the inclusion and exclusion criteria. IDH, isocitrate dehydrogenase.

In this retrospective study, patients were recruited between April 2010 and March 2022 from the Department of Neuroradiology of the University Hospital of Heidelberg (Germany). A total of 451 patients with pretreatment IDH wild-type glioblastoma were included if the following criteria were met: i) the diagnosis of glioblastoma was confirmed by pathology, ii) high-quality MRI data of pre- and post-contrast T1-weighted (T1w and T1-CE, respectively) were available, T2-weighted (T2w) and fluid attenuated inversion recovery (FLAIR) images were available prior to treatment, and (iii) medical reports with epilepsy status at diagnosis were available. Here, the epilepsy status at diagnosis was mostly based on clinical presentation or anamnestic information provided by patients. **Figure 1** shows the flow diagram of the study population and summarizes the exclusion criteria. The retrospective evaluation of de-identified imaging and medical data was approved by the Ethics Committee of the University of Heidelberg.

### Magnetic resonance imaging

As presented in another work (13), every patient in this retrospective study received MR imaging of their head in one of the following 3.0-T clinical magnetic resonance devices: Magnetom Trio TIM, Prisma fit, Verio, or Skyra (all from Siemens Healthineers AG, Germany). For signal acquisition, a 12-channel head-matrix coil was used. The MR protocol included the following four sequences: axial T1w and T1-CE images after the administration of a gadolinium-based contrast agent, an axial T2w image, and an axial FLAIR image. For obtaining T1w and T1-CE, a 3D magnetization-prepared rapid acquisition with gradient echo (MPRAGE) sequence was used with the following parameters: inversion time (TI) = 900–1,100 ms, echo time (TE) = 3–4 ms, repetition time (TR) = 1,710–2,250 ms, and flip angle = 15°. Gadoterate meglumine (Dotarem^®^, Guerbet, France) with a dose of 0.1 mmol/kg was administered as an MR contrast agent. For axial T2, the following parameters were chosen: TE = 85–88 ms, TR = 2,740–5,950 ms, section thickness = 5 mm, and spacing = 5.5 mm. For axial FLAIR, the parameters were as follows: TI = 2,400–2,500 ms, TE = 85–135 ms, TR = 8,500–10,000 ms, section thickness = 5 mm, and spacing = 5.5 mm.

### Image preprocessing—brain extraction, intensity normalization, registration, and segmentation

HD-BET was used as a semi-automatic brain extraction tool to obtain brain images from each MRI (14). T2w, T1-CE, and FLAIR were registered to T1w images of the same MRI exam by using FSL-FLIRT (15, 16). MRI signal intensity values were normalized using white-stripe normalization as described here (17). CET regions in T1-CE images and non-enhancing lesion (NEL) presenting as T2w-/FLAIR-hyperintense regions in FLAIR images were segmented automatically using HD-GLIO (18, 19). Normal-appearing WM was segmented by using FAST (20) from the FSL software library (version 6.0, Oxford, United Kingdom) (21). After a visual inspection of segmented masks obtained from HD-GLIO by two board-certified neuroradiologists (J.M.E.J., F.T.K.), the segmentation masks were manually corrected in consensus if the segmentation was inaccurate. Corrections for the segmentation were needed for 21 cases (<5% of total cases). Furthermore, a 5-mm layer surrounding the CET was identified as a peritumoral region (PeriCET). Masks for these peritumoral regions were obtained using five iterations of the binary_dilation-function from Python’s SciPy package (SciPy 1.12.0).

### Radiomics feature extraction

For radiomics feature extraction, PyRadiomics 3.1.0 was used (22). For each ROI, 4 * 19 first-order features, 14 2D and 3D shape features, and 4 * 75 texture features were extracted from T1w, T2w, T1-CE, and FLAIR images without applying any image filters. Therefore, a total of up to 390 radiomics features were extracted from a single ROI. Due to high computing time, shape features were not extracted from normal-appearing white matter masks. Texture features were included from the following feature classes: gray-level co-occurrence matrix, gray-level run length matrix, gray-level size zone matrix, gray-level dependence matrix, and neighborhood gray-tone difference matrix. A complete list of radiomics features can be obtained from the supplementary materials (see **Supplementary Table S1**). Details about the radiomics features were described in the PyRadiomics documentation (https://pyradiomics.readthedocs.io/en/latest/).

### Machine learning classification

The dataset was randomly split into a training cohort (*n* = 315, 70%) and a validation cohort (*n* = 136, 30%). Here, the epilepsy status at diagnosis was used to stratify both cohorts to obtain two cohorts with equal frequency of positive/negative epilepsy status.

The following steps were performed with the training cohorts. First, the feature selection technique using ElasticNet was used, which can be seen as a generalization of the Lasso technique (23). Then, a machine learning model was trained with the selected radiomics features. For the machine learning model, logistic regression (LR), support vector machine with a linear kernel (L-SVM), and a neural network as a multilayer perceptron classifier (MLPC) were used. To determine appropriate parameters for the feature selection (ElasticNet) and the machine learning model, hyperparameter optimizations were performed using the grid-search cross-validation technique (GridSearchCV) with a stratified 10-fold cross-validation with 10 repetitions. A more detailed list of parameters chosen for each model can be obtained from the supplementary materials (see **Supplementary Table S2**).

Finally, the performance of the optimized model was evaluated with the validation cohort, which was neither used for training the model nor for the hyperparameter optimization in previous steps. The area under the receiver operating characteristic curve (AUC) was used as a metric to evaluate the model’s performance.

To get insights into the most relevant radiomics features for the best performing machine learning model, permutation feature importance was computed with 1,000 repetitions. Here, the loss of a model’s performance after randomly shuffling a single feature was used as an indicator of the importance for the model’s prediction (24). For our analysis, every value of permutation feature importance was normalized to the maximum value of 100 and a minimum value of 0 as described here (25).

All machine learning steps were performed in Python 3.10.12 (Python Software Foundation, Delaware, USA) using scikit-learn 1.4.1.

### Statistical analysis

The 95% confidence interval (CI) of AUC was computed and compared according to DeLong’s method (26) implemented in R version 4.3.1 (The R Foundation for Statistical Computing, Vienna, Austria). GraphPad Prism 10.0.2 (GraphPad Software Inc., Boston, USA) and Python 3.10.12 (Python Software Foundation, Delaware, USA) were used to compare patient characteristics using the two-sided Mann–Whitney test for age at diagnosis and Fisher’s exact tests for sex and epilepsy status. A difference was reported as significant if the *p*-value from the statistical test was less than 0.05.

## Results

### Patient characteristics

Patient characteristics from the training and validation cohorts can be obtained from **Table 1**. The mean age and standard deviation were (61.2 ± 11.8) years. In this study, 268 patients (59.4%) were men, and 183 patients (40.6%) were women. The training and validation cohorts were not different regarding age at diagnosis (two-sided Mann–Whitney test, *p* = 0.43), sex (two-sided Fisher’s exact test, *p* = 0.75), and epilepsy status (two-sided Fisher’s exact test, *p* = 1.0).

**Table 1:** Patient characteristics from the training and validation cohorts.

Parameters		Training cohort (*n* = 315, 70%)	Validation cohort (*n* = 136, 30%)	*p*-value
Age	In years	61.3 ± 12.1	61.0 ± 11.0	0.4383
Sex	Male	189	79	0.7541
	Female	126	57	
Epilepsy status	With epilepsy	80	35	1.0
	Without epilepsy	235	101	

For age, means ± standard deviation and *p*-value from a Mann–Whitney test are presented. For sex and epilepsy status, *p*-values are obtained from Fisher’s exact tests.

### Machine learning models

**Figure 2:**
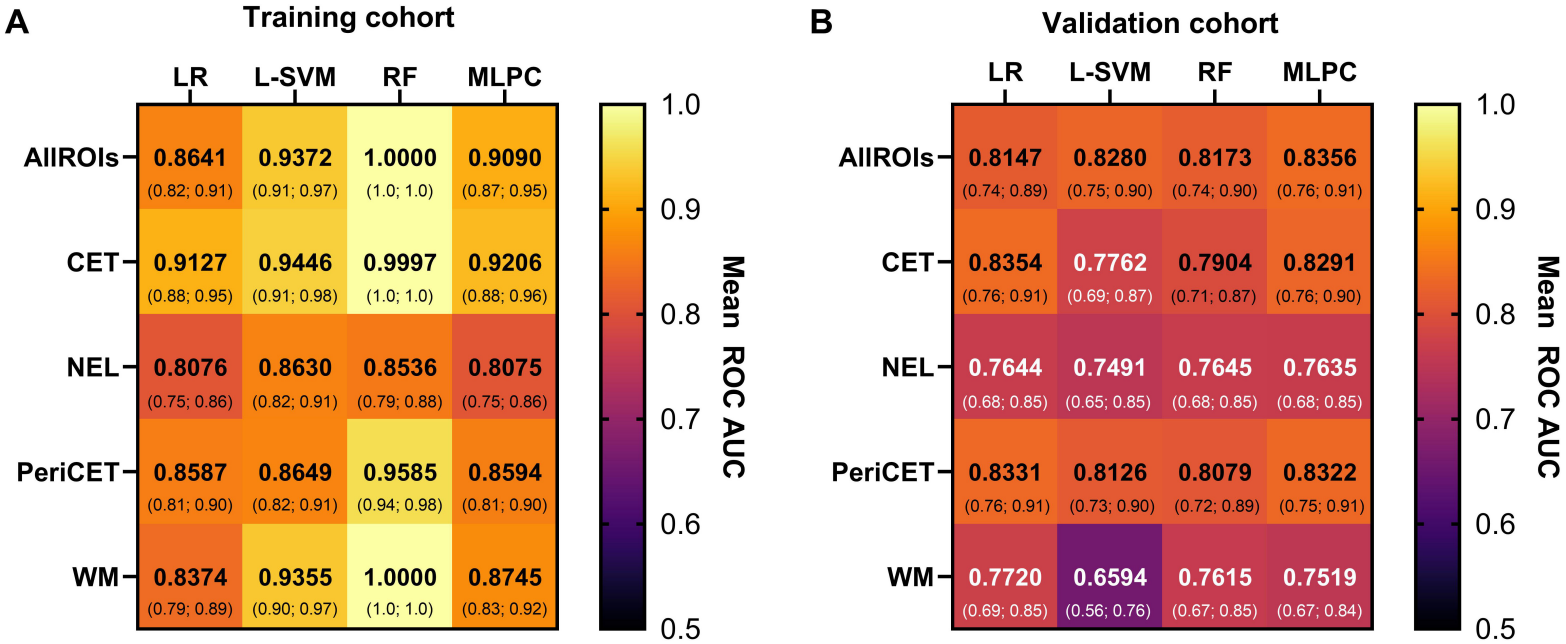
The performance of the machine learning model with data from the **(A)** training cohort and **(B)** validation cohort is summarized here. Mean AUC values with 95% confidence intervals (below) are shown for each combination of models [logistic regression (LR), linear support vector machine (L-SVM), random forest (RF), multilayer perceptron classifier (MLPC)] and ROIs [contrast-enhancing tumor (CET), non-enhancing lesion (NEL), peritumoral region (PeriCET), white matter (WM)].

**Figure 3:**
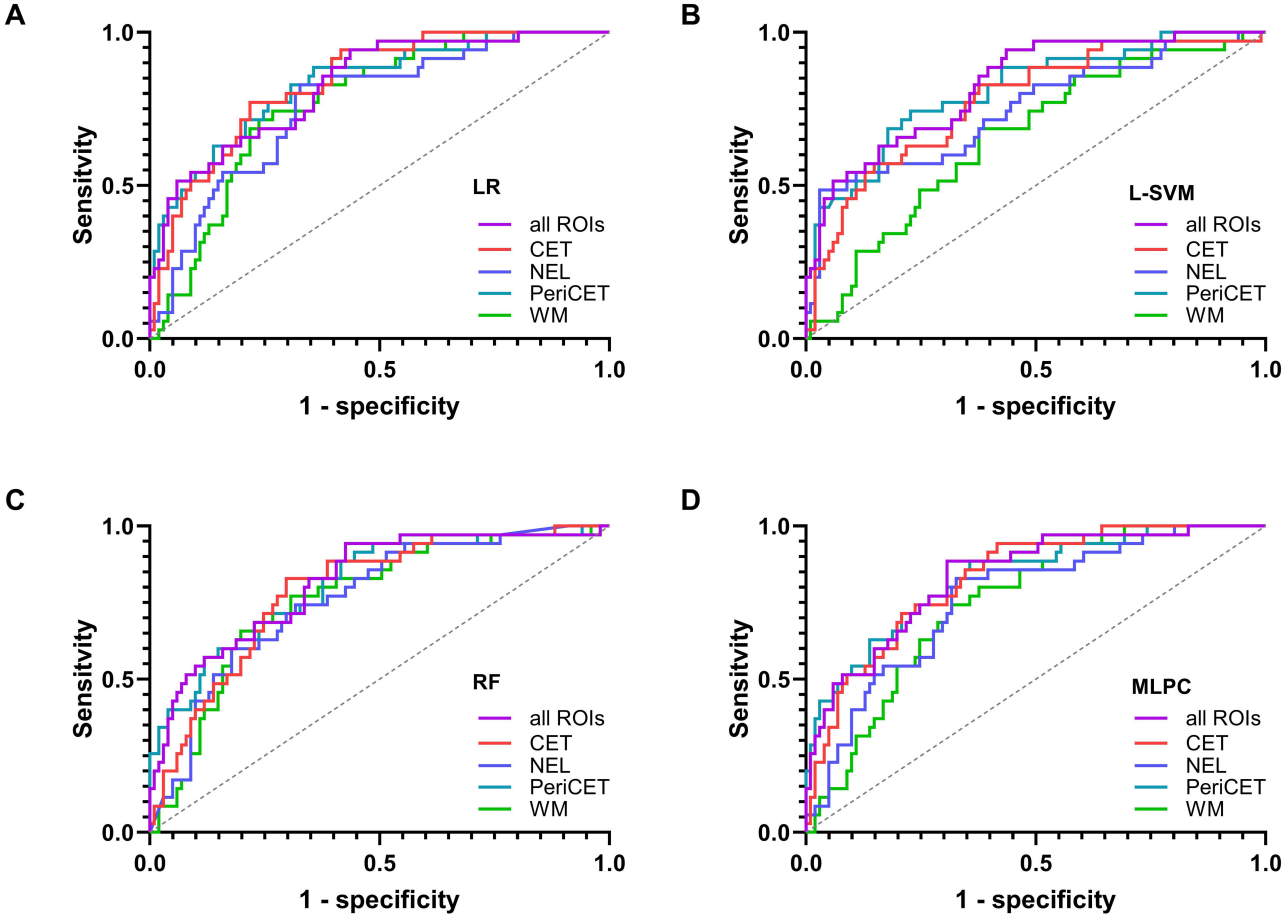
The receiver operating characteristic curves for the validation cohort are shown for each model in **(A)** logistic regression (LR), **(B)** support vector machine with linear kernel (L-SVM), **(C)** random forest (RF), and **(D)** multilayer perceptron classifier (MLPC).

Machine learning models could be trained with radiomics features from a single ROI or a combination of all ROIs (i.e., CET, NEL, PeriCET, and WM) using data from the training cohort. The performance levels of all these trained models to identify epilepsy status for patients in the training cohort and validation cohort are summarized in **Figure 2** as a heat map. A table representation of these results can be found in the supplementary materials (see **Supplementary Table S3**). The corresponding receiver operating characteristic curves for the validation cohort are presented in **Figure 3**.

The performance levels of machine learning models utilizing radiomics features from all available ROIs used in this study (i.e., CET, NEL, PeriCET, and WM) were consistent regardless of the choice of the model. The best performing model here was achieved by using MLPC with a mean AUC of 0.8356 (95% CI: [0.7609; 0.9104]) for the validation cohort.

Similar performance levels for the validation cohort were observed if the models utilized only radiomics features from the tumor (CET) or peritumoral (PeriCET) regions. For radiomics features extracted only from PeriCET, the best performing model to identify epilepsy status in the validation cohort was achieved using LR with a mean AUC of 0.8331 (95% CI: [0.7558; 0.9104]). A similar performance could be obtained by using other models: L-SVM (mean AUC: 0.8126, 95% CI: [0.7284; 0.8968]), random forest (RF) (mean AUC: 0.8079, 95% CI: [0.7234; 0.8924]), and MLPC (mean AUC: 0.8322, 95% CI: [0.7546; 0.9099]).

For radiomics features extracted only from WM masks, the best performing model to identify epilepsy status in the validation cohort was achieved by using LR with a mean AUC of 0.7720 (95% CI: [0.6903; 0.8536]). A similar performance could be obtained by using RF (mean AUC: 0.7615, 95% CI: [0.6716; 0.8514]) and MLPC (mean AUC: 0.7519, 95% CI: [0.6680; 0.8359]). L-SVM using only radiomics features from WM masks had the worst performance in our analysis, with a mean AUC of 0.6594 (95% CI: [0.5579; 0.7609]), although the mean AUC for the training cohort was 0.9355 (95% CI: [0.8980; 0.9731]).

### Feature importance

**Figure 4:**
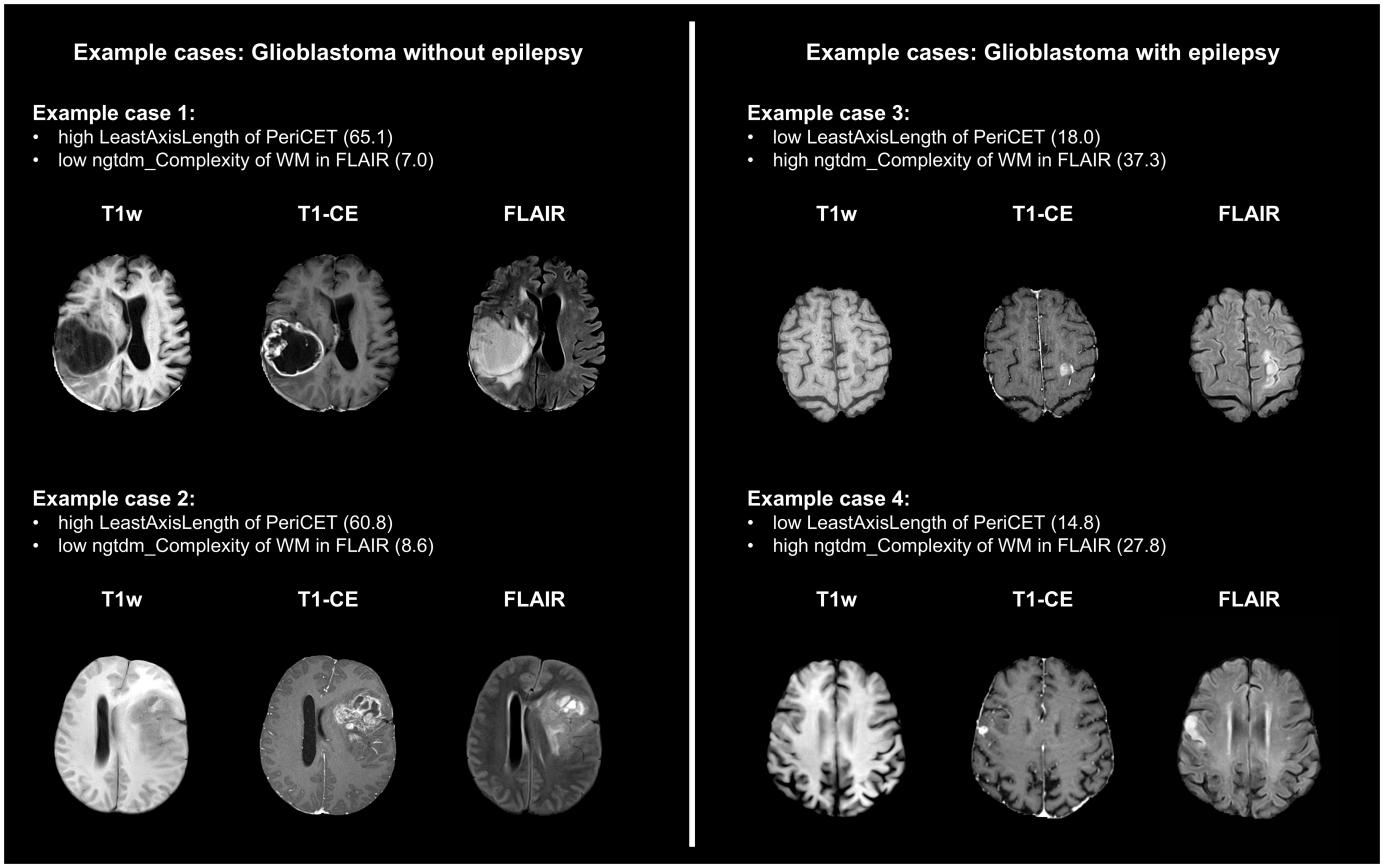
Four example cases are presented here with prominent values for radiomics features, which were important for the classification of epilepsy-associated glioblastoma. LeastAxisLength was the most important feature for models trained with only features from the peritumor region (PeriCET). For the white matter region (WM), complexity from neighborhood gray-tone difference matrix (ngtdm) in FLAIR was the most relevant feature.

The five most relevant radiomics features are listed in **Table 2** in descending order of the feature importance values obtained from the best performing model. A complete list of features with the normalized scores can be found in the supplementary materials (see **Supplementary Tables S4**-**S8** as Excel spreadsheets). If radiomics features from all ROIs were available for the machine learning model, mostly shape features from tumor and peritumor ROIs were relevant. For the LR model, which was trained exclusively on radiomics features extracted from the CET region, higher-order features from T1-CE, T1w, and FLAIR were among the five most important features. For peritumoral regions, both NEL and PeriCET, first-order features from FLAIR images and shape features were the most relevant features to identify epilepsy status in the validation cohort. Here, shape features related to axis length, like Minor- and MajorAxisLength from NEL and LeastAxisLength and Maximum2DDiameterSlice from PeriCET, were shown to be most relevant. Higher-order features from WM regions in FLAIR images were identified as the most important radiomics features for the model if only features from WM regions were used for the classification task. However, the 10th percentile of white-striped normalized intensity values from the white-striped normalized T2w was also shown to be highly relevant for identifying glioblastoma patients with epileptic seizure at pretreatment. In **Figure 4**, example cases are shown with prominent values of radiomics features, which were most relevant for the classification.

**Table 2:** The five most relevant radiomics features obtained from permutation feature importance values from the best performing model in the validation cohort.

Best performing model	Radiomics feature	Feature importance
All ROIs = CET + NEL + PeriCET + WM		
MLPC	CET_T1w_glszm_LowGrayLevelZoneEmphasis	100
	CET_FLAIR_ngtdm_Coarseness	99.8
	CET_shape_LeastAxisLength	81.1
	CET_shape_SurfaceArea	71.6
	PeriCET_FLAIR_firstorder_Kurtosis	69.3
CET		
LR	CET_T1w_gldm_SmallDependenceLowGrayLevelEmphasis	100
	CET_T1-CE_ngtdm_Strength	76.6
	CET_FLAIR_gldm_SmallDependenceLowGrayLevelEmphasis	57.9
	CET_T1w_glszm_LowGrayLevelZoneEmphasis	53.4
	CET_FLAIR_firstorder_Skewness	41.8
NEL		
RF	NEL_shape_MinorAxisLength	100
	NEL_shape_MajorAxisLength	78.7
	NEL_shape_Maximum2DDiameterColumn	51.8
	NEL_FLAIR_firstorder_10Percentile	36.4
	NEL_shape_SurfaceArea	21.8
PeriCET		
LR	PeriCET_shape_LeastAxisLength	100
	PeriCET_shape_Maximum2DDiameterSlice	68.8
	PeriCET_shape_SurfaceArea	62.7
	PeriCET_FLAIR_firstorder_Kurtosis	51.3
	PeriCET_FLAIR_firstorder_Minimum	47.3
WM		
LR	WM_FLAIR_ngtdm_Complexity	100
	WM_T2_firstorder_10Percentile	97.3
	WM_FLAIR_gldm_SmallDependenceHighGrayLevelEmphasis	90.8
	WM_FLAIR_glszm_ZonePercentage	67.0
	WM_FLAIR_gldm_SmallDependenceEmphasis	57.4

The permutation feature importance values presented here are normalized to the maximum value of feature importance as 100.

CET, contrast-enhancing tumor; NEL, non-enhancing lesion; PeriCET, peritumoral region; LR, logistic regression; RF, random forest; MLPC, multilayer perceptron classifier; glszm, gray-level size zone matrix; gldm, gray-level dependence matrix; ngtdm, neighborhood gray-tone difference matrix.

## Discussion

In this retrospective study with a total of 451 pretreatment glioblastoma patients, radiomics features extracted from conventional brain MRIs were assessed for potential associations with pretreatment epilepsy status. In the approach chosen, the ROI was not limited to the CET or the NEL presenting as hyperintense signal in T2w/FLAIR images. Instead, radiomics features from the 5-mm layer of the peritumoral region surrounding the CET (PeriCET) and the normal-appearing WM region were investigated as well. It could be demonstrated that machine learning models like logistic regression and support vector machines can identify epilepsy status of pretreatment glioblastoma with radiomics features obtained only from the PeriCET (mean AUC: 0.83) or the WM regions (mean AUC: 0.77) with good predictive performance levels. For PeriCET, the predictive performance of the model was similar to the models using features only from the CET or a combination of all ROIs and was superior to the models using features only from the NEL and WM.

Classifiers such as LR, RF, and SVM differ substantially in how they process feature interactions, manage redundancy, and cope with noise (27). While RF and SVM are better able to capture more complex structures within a dataset than simpler classifiers like LR as demonstrated in a radiomics study about glioma grading (28), RF and SVM can be vulnerable to overfitting, limiting the overall performance of the model. There are multiple other studies about radiomics in the context of neuro-oncology (25, 29, 30), and we observed similar performance levels of the models across different classifiers. Furthermore, permutation-based feature importance tends to highlight the features best utilized by each specific model, rather than identifying a universally optimal subset. The limited overlap in top-ranked features across models reflects the high-dimensional, non-convex nature of the radiomics domain, where multiple distinct solutions can capture different aspects of the underlying biological variance. For instance, shape-based features emerged as highly predictive within the tumor core and peritumoral regions, likely reflecting the pronounced morphological distortions typical of these zones. In contrast, higher-order textural features played a dominant role in classifying white matter regions, where the baseline tissue structure is relatively homogeneous but may exhibit subtle signal variations, particularly in patients predisposed to epileptogenic changes. Similarly, as shown in another study, shape features from peritumor region and texture features from white matter regions were relevant to identify progressive glioblastoma at post-treatment (31). These findings underscore the idea that each ROI contributes distinct, non-redundant information of biological relevance. Different models appear to exploit this heterogeneity in complementary ways, depending on their architectural properties and regularization strategies. Importantly, the ability of different models to achieve similar performance using distinct feature sets has positive implications for generalizability. It suggests that the radiomic signature associated with epilepsy status in glioblastoma was not dependent on any one model or feature subset, but rather was distributed across multiple, semantically meaningful feature domains. This redundancy enhances the robustness of the findings and reduces the likelihood that observed performance is an artifact of overfitting to a specific algorithmic bias.

Since radiomics allows a more quantitative evaluation of radiological data, the radiomics approach can offer a powerful tool for clinicians to gain valuable information regarding molecular and clinical characteristics associated with tumor disease in a non-invasive manner (32). In this study, radiomics features could be identified and correlated with epilepsy status of pretreatment glioblastoma patients in a large cohort. Our results can potentially contribute to better identifying these patients at risk of developing epilepsy. Furthermore, it underlines the important role of the peritumoral region for developing epilepsy in neuro-oncology patients.

Multiple works have been published where MRI-based radiomics features were used to predict epilepsy associated with adult diffuse glioma (33–36). Two studies included only patients with low-grade glioma (33, 34). Wang et al. enrolled 205 patients retrospectively with only low-grade glioma to investigate the role of radiomics features for identifying the epilepsy type (34). In a study with 286 low-grade glioma patients, Liu et al. developed models with radiomics features extracted from NEL to predict patients’ epilepsy status at diagnosis (AUC = 0.82 with the validation cohort) (33). In two other studies, a mixture of low- and high-grade glioma was included in their analysis (35, 36). Both studies reported good performances of their machine learning models to identify epilepsy status in their validation cohorts with AUC values of 0.836 and 0.866 from Gao et al. (35) and Jie et al. (36), respectively. Here, Gao et al. included 166 patients with frontal glioma, while Jie et al. included a total of 380 low- and high-grade glioma patients. Since epileptic seizures are much more frequently observed in patients with low-grade glioma than with high-grade glioma (90% vs. 30%–50%) (3, 37), analyses from Gao et al. and Jie et al. may rather reflect the glioma grading instead of the epilepsy status. It has already been demonstrated by various groups that machine learning or deep learning approaches with MR-based radiomics features can reveal glioma grading with very good predictive performance levels (6, 38, 39). Therefore, analyzing low- and high-grade glioma patients separately may be more appropriate to identify radiomics signatures specifically associated with epilepsy status. In our study, we investigated a larger patient cohort (*n* = 451) with only glioblastoma. The IDH-wild type was confirmed here by pathology for each patient. With machine learning models, we achieved similar predictive performance with glioblastoma patients as reported by Liu et al., who investigated a patient cohort with low-grade glioma only. In another work, we could show that glioblastoma with epilepsy at diagnosis was associated with less tumor burden than without epilepsy. As already discussed there, this might be the result from earlier diagnosis of epilepsy-associated glioblastoma (40). The prominent role of shape features from tumor and peritumor regions in our study here supports our previous findings.

This retrospective study has several limitations. First, the epilepsy status at diagnosis was mostly based on clinical presentation or anamnestic information provided by patients. Further information regarding the diagnosis of epilepsy, for example, results from electroencephalogram, was not available. Second, omitting shape features from the normal-appearing white matter region may have impacted the comparative richness of features between ROIs. However, these features were deliberately excluded from the white matter ROI due to the absence of discrete lesion boundaries in WM masks. Furthermore, we have not investigated models trained with any combinations of radiomics features extracted from two or three different ROIs, which might result in more powerful classifiers. Including multiple ROIs increases the dimensionality of the feature space, which may introduce redundancy and therefore be more vulnerable to overfitting. In our study, overfitting could be even observed for models trained with radiomics features from a single ROI. That is why we believe that combining radiomics features from two or three ROIs might not necessarily result in improved models. Finally, the monocentric study design may limit the generalizability of the study results. A multicentric study design with various MRI devices may result in more reproducible radiomics features. As investigated and discussed by multiple other groups (8, 41), MRI-based radiomics features can be influenced by the choice of measurement parameters. In our study, various clinical MR machines with a wide range of TR for T2w images and TE for FLAIR sequences were used for image acquisitions. This can introduce heterogeneity that can enhance the robustness of machine learning models by exposing them to a broader range of imaging characteristics during training.

## Conclusion

Radiomics features from conventional MRIs of the brain can predict the epilepsy status for pretreatment glioblastoma patients. Machine learning models were trained and validated with radiomics features extracted from various ROIs from a large patient cohort. Here, the ROI was not limited to the CET and NEL but included the peritumoral region (PeriCET) and normal-appearing WM. The consistent predictive performance of the models using only radiomics features from PeriCET or WM underlines the important role of these brain regions for developing epilepsy associated with glioblastoma.

## Data Availability

The datasets presented in this article are not readily available because the de-identified data that support the findings of this study are available from the corresponding author upon reasonable request. Requests to access the datasets should be directed to YCY, YeongChul.Yun@med.uni-heidelberg.de.

## References

[1] Ostrom QT Price M Neff C Cioffi G Waite KA Kruchko C. Cbtrus statistical report: primary brain and other central nervous system tumors diagnosed in the United States in 2016—2020. Neuro Oncol. (2023) 25:iv1–iv99. doi: 10.1093/neuonc/noad149, PMID: 37793125 PMC10550277

[2] Omuro A DeAngelis LM. Glioblastoma and other Malignant gliomas: A clinical review. JAMA. (2013) 310:1842–50. doi: 10.1001/jama.2013.280319, PMID: 24193082

[3] van Breemen MSM Wilms EB Vecht CJ. Epilepsy in patients with brain tumours: epidemiology, mechanisms, and management. Lancet Neurol. (2007) 6:421–30. doi: 10.1016/S1474-4422(07)70103-5, PMID: 17434097

[4] Kumar V Gu Y Basu S Berglund A Eschrich SA Schabath MB. Radiomics: the process and the challenges. Magn Reson Imaging. (2012) 30:1234–48. doi: 10.1016/j.mri.2012.06.010, PMID: 22898692 PMC3563280

[5] Zwanenburg A Vallières M Abdalah MA Aerts HJWL Andrearczyk V Apte A. The image biomarker standardization initiative: standardized quantitative radiomics for high-throughput image-based phenotyping. Radiology. (2020) 295:328–38. doi: 10.1148/radiol.2020191145, PMID: 32154773 PMC7193906

[6] Kobayashi K Miyake M Takahashi M Hamamoto R. Observing deep radiomics for the classification of glioma grades. Sci Rep. (2021) 11:10942. doi: 10.1038/s41598-021-90555-2, PMID: 34035410 PMC8149679

[7] Singh G Manjila S Sakla N True A Wardeh AH Beig N. Radiomics and radiogenomics in gliomas: A contemporary update. Br J Cancer. (2021) 125:641–57. doi: 10.1038/s41416-021-01387-w, PMID: 33958734 PMC8405677

[8] Fan H Luo Y Gu F Tian B Xiong Y Wu G. Artificial intelligence-based mri radiomics and radiogenomics in glioma. Cancer Imaging. (2024) 24:36. doi: 10.1186/s40644-024-00682-y, PMID: 38486342 PMC10938723

[9] Baayen JC de Jongh A Stam CJ de Munck JC Jonkman JJ Kasteleijn-Nolst Trenité DGA. Localization of slow wave activity in patients with tumor-associated epilepsy. Brain Topogr. (2003) 16:85–93. doi: 10.1023/B:BRAT.0000006332.71345.b7, PMID: 14977201

[10] Köhling R Senner V Paulus W Speckmann E-J. Epileptiform activity preferentially arises outside tumor invasion zone in glioma xenotransplants. Neurobiol Dis. (2006) 22:64–75. doi: 10.1016/j.nbd.2005.10.001, PMID: 16309916

[11] You G Sha Z Jiang T. The pathogenesis of tumor-related epilepsy and its implications for clinical treatment. Seizure. (2012) 21:153–9. doi: 10.1016/j.seizure.2011.12.016, PMID: 22300623

[12] de Groot M Reijneveld JC Aronica E Heimans JJ. Epilepsy in patients with a brain tumour: focal epilepsy requires focused treatment. Brain. (2012) 135:1002–16. doi: 10.1093/brain/awr310, PMID: 22171351

[13] Kickingereder P Neuberger U Bonekamp D Piechotta PL Götz M Wick A. Radiomic subtyping improves disease stratification beyond key molecular, clinical, and standard imaging characteristics in patients with glioblastoma. Neuro Oncol. (2018) 20:848–57. doi: 10.1093/neuonc/nox188, PMID: 29036412 PMC5961168

[14] Isensee F Schell M Pflueger I Brugnara G Bonekamp D Neuberger U. Automated brain extraction of multisequence mri using artificial neural networks. Hum Brain Mapp. (2019) 40:4952–64. doi: 10.1002/hbm.24750, PMID: 31403237 PMC6865732

[15] Jenkinson M Smith S. A Global Optimisation Method for Robust Affine Registration of Brain Images. Med Image Anal. (2001) 5:143–56. doi: 10.1016/S1361-8415(01)00036-6, PMID: 11516708

[16] Jenkinson M Bannister P Brady M Smith S. Improved optimization for the robust and accurate linear registration and motion correction of brain images. Neuroimage. (2002) 17:825–41. doi: 10.1006/nimg.2002.1132, PMID: 12377157

[17] Shinohara RT Sweeney EM Goldsmith J Shiee N Mateen FJ Calabresi PA. Statistical normalization techniques for magnetic resonance imaging. NeuroImage Clin. (2014) 6:9–19. doi: 10.1016/j.nicl.2014.08.008, PMID: 25379412 PMC4215426

[18] Isensee F Jaeger PF Kohl SAA Petersen J Maier-Hein KH. Nnu-net: A self-configuring method for deep learning-based biomedical image segmentation. Nat Methods. (2021) 18:203–11. doi: 10.1038/s41592-020-01008-z, PMID: 33288961

[19] Kickingereder P Isensee F Tursunova I Petersen J Neuberger U Bonekamp D. Automated quantitative tumour response assessment of mri in neuro-oncology with artificial neural networks: A multicentre, retrospective study. Lancet Oncol. (2019) 20:728–40. doi: 10.1016/S1470-2045(19)30098-1, PMID: 30952559

[20] Zhang Y Brady M Smith S. Segmentation of brain mr images through a hidden markov random field model and the expectation-maximization algorithm. IEEE Trans Med Imaging. (2001) 20:45–57. doi: 10.1109/42.906424, PMID: 11293691

[21] Jenkinson M Beckmann CF Behrens TEJ Woolrich MW Smith SM. Fsl. Neuroimage. (2012) 62:782–90. doi: 10.1016/j.neuroimage.2011.09.015, PMID: 21979382

[22] van Griethuysen JJM Fedorov A Parmar C Hosny A Aucoin N Narayan V. Computational radiomics system to decode the radiographic phenotype. Cancer Res. (2017) 77:e104–e7. doi: 10.1158/0008-5472.CAN-17-0339, PMID: 29092951 PMC5672828

[23] Zou H Hastie T. Regularization and variable selection via the elastic net. J R Stat Soc Ser B Stat Methodol. (2005) 67:301–20. doi: 10.1111/j.1467-9868.2005.00503.x

[24] Breiman L. Random forests. Mach Learn. (2001) 45:5–32. doi: 10.1023/A:1010933404324

[25] Bae S An C Ahn SS Kim H Han K Kim SW. Robust performance of deep learning for distinguishing glioblastoma from single brain metastasis using radiomic features: model development and validation. Sci Rep. (2020) 10:12110. doi: 10.1038/s41598-020-68980-6, PMID: 32694637 PMC7374174

[26] DeLong ER DeLong DM Clarke-Pearson DL. Comparing the areas under two or more correlated receiver operating characteristic curves: A nonparametric approach. Biometrics. (1988) 44:837–45. doi: 10.2307/2531595 3203132

[27] Perniciano A Loddo A Di Ruberto C Pes B. Insights into radiomics: impact of feature selection and classification. Multimedia Tools Appl. (2024) 84: 31695–721. doi: 10.1007/s11042-024-20388-4

[28] Sun P Wang D Mok VC Shi L. Comparison of feature selection methods and machine learning classifiers for radiomics analysis in glioma grading. IEEE Access. (2019) 7:102010–20. doi: 10.1109/ACCESS.2019.2928975

[29] Chen C Zheng A Ou X Wang J Ma X. Comparison of radiomics-based machine-learning classifiers in diagnosis of glioblastoma from primary central nervous system lymphoma. Front Oncol. (2020) 10:1151. doi: 10.3389/fonc.2020.01151, PMID: 33042784 PMC7522159

[30] Wu S Meng J Yu Q Li P Fu S. Radiomics-based machine learning methods for isocitrate dehydrogenase genotype prediction of diffuse gliomas. J Cancer Res Clin Oncol. (2019) 145:543–50. doi: 10.1007/s00432-018-2787-1, PMID: 30719536 PMC6394679

[31] Yun YC Jende JME Garhöfer F Wolf S Holz K Hohmann A. Combined peritumoral radiomics and clinical features predict 12-month progression free survival in glioblastoma. J Neurooncol. (2025) 174: 111–120. doi: 10.1007/s11060-025-05037-6, PMID: 40244521

[32] Lambin P Leijenaar RTH Deist TM Peerlings J de Jong EEC van Timmeren J. Radiomics: the bridge between medical imaging and personalized medicine. Nat Rev Clin Oncol. (2017) 14:749–62. doi: 10.1038/nrclinonc.2017.141, PMID: 28975929

[33] Liu Z Wang Y Liu X Du Y Tang Z Wang K. Radiomics analysis allows for precise prediction of epilepsy in patients with low-grade gliomas. NeuroImage Clin. (2018) 19:271–8. doi: 10.1016/j.nicl.2018.04.024, PMID: 30035021 PMC6051495

[34] Wang Y Wei W Liu Z Liang Y Liu X Li Y. Predicting the type of tumor-related epilepsy in patients with low-grade gliomas: A radiomics study. Front Oncol. (2020) 10:235. doi: 10.3389/fonc.2020.00235, PMID: 32231995 PMC7082349

[35] Gao A Yang H Wang Y Zhao G Wang C Wang H. Radiomics for the prediction of epilepsy in patients with frontal glioma. Front Oncol. (2021) 11:725926. doi: 10.3389/fonc.2021.725926, PMID: 34881174 PMC8645689

[36] Jie B Hongxi Y Ankang G Yida W Guohua Z Xiaoyue M. Radiomics nomogram improves the prediction of epilepsy in patients with gliomas. Front Oncol. (2022) 12:856359. doi: 10.3389/fonc.2022.856359, PMID: 35433444 PMC9007085

[37] Pallud J McKhann GM. Diffuse low-grade glioma-related epilepsy. Neurosurg Clin N Am. (2019) 30:43–54. doi: 10.1016/j.nec.2018.09.001, PMID: 30470404

[38] Hashido T Saito S Ishida T. Radiomics-based machine learning classification for glioma grading using diffusion- and perfusion-weighted magnetic resonance imaging. J Comput Assist Tomogr. (2021) 45: 606–13. doi: 10.1097/RCT.0000000000001180, PMID: 34270479

[39] Malik N Geraghty B Dasgupta A Maralani PJ Sandhu M Detsky J. Mri radiomics to differentiate between low grade glioma and glioblastoma peritumoral region. J Neurooncol. (2021) 155:181–91. doi: 10.1007/s11060-021-03866-9, PMID: 34694564

[40] Yun YC Wolf S Holz K Garhöfer F Hohmann A Vollmuth P. Mapping glioblastoma-induced neurological deficits: A brain atlas. Clin Neurol Neurosurg. (2025) 253:108911. doi: 10.1016/j.clineuro.2025.108911, PMID: 40253841

[41] Molina D Pérez-Beteta J Martínez-González A Martino J Velasquez C Arana E. Lack of robustness of textural measures obtained from 3d brain tumor mris impose a need for standardization. PloS One. (2017) 12:e0178843. doi: 10.1371/journal.pone.0178843, PMID: 28586353 PMC5460822

