# Supplementary material for: Radiomics features from the peritumoral region can be associated with the epilepsy status of glioblastoma patients
Source: Front Oncol. 2025 Aug 25;15:1587745. doi: 10.3389/fonc.2025.1587745 (PMC12416087; doi:10.3389/fonc.2025.1587745)
Supplement: Supplementary file 1 [file DataSheet1.pdf]

Radiomic features from the peritumoral region can be associated with epilepsy status of glioblastoma patients

Supplementary Materials

Table S1: Complete list of radiomics features extracted with PyRadiomics grouped by feature classes.

Feature class	Feature names
First order features<br>(n=19)	Energy<br>TotalEnergy<br>Entropy<br>Minimum<br>10Percentile<br>90Percentile<br>Maximum<br>Mean<br>Median<br>InterquartileRange<br>Range<br>MeanAbsoluteDeviation<br>RobustMeanAbsoluteDeviation<br>RootMeanSquared<br>StandardDeviation<br>Skewness<br>Kurtosis<br>Variance<br>Uniformity
Shape features<br>(n=14)	MeshVolume<br>VoxelVolume<br>SurfaceArea<br>SurfaceVolumeRatio<br>Sphericity<br>Maximum3DDiameter<br>Maximum2DDiameterSlice<br>Maximum2DDiameterColumn<br>Maximum2DDiameterRow<br>MajorAxisLength<br>MinorAxisLength<br>LeastAxisLength<br>Elongation<br>Flatness
Texture features from GLCM<br>(n=24)	glcm_Autocorrelation<br>glcm_JointAverage<br>glcm_ClusterProminence<br>glcm_ClusterShade<br>glcm_ClusterTendency<br>glcm_Contrast<br>glcm_Correlation<br>glcm_DifferenceAverage<br>glcm_DifferenceEntropy<br>glcm_DifferenceVariance<br>glcm_JointEnergy<br>glcm_JointEntropy<br>glcm_Imc1

	glcm_Imc2<br>glcm_Idm<br>glcm_MCC<br>glcm_Idmn<br>glcm_Id<br>glcm_Idn<br>glcm_InverseVariance<br>glcm_MaximumProbability<br>glcm_SumAverage<br>glcm_SumEntropy<br>glcm_SumSquares
Texture features from GLSZM<br>(n=16)	glszm_SmallAreaEmphasis<br>glszm_LargeAreaEmphasis<br>glszm_GrayLevelNonUniformity<br>glszm_GrayLevelNonUniformityNormalized<br>glszm_SizeZoneNonUniformity<br>glszm_SizeZoneNonUniformityNormalized<br>glszm_ZonePercentage<br>glszm_GrayLevelVariance<br>glszm_ZoneVariance<br>glszm_ZoneEntropy<br>glszm_LowGrayLevelZoneEmphasis<br>glszm_HighGrayLevelZoneEmphasis<br>glszm_SmallAreaLowGrayLevelEmphasis<br>glszm_SmallAreaHighGrayLevelEmphasis<br>glszm_LargeAreaLowGrayLevelEmphasis<br>glszm_LargeAreaHighGrayLevelEmphasis
Texture features from GLRLM<br>(n=16)	glrlm_ShortRunEmphasis<br>glrlm_LongRunEmphasis<br>glrlm_GrayLevelNonUniformity<br>glrlm_GrayLevelNonUniformityNormalized<br>glrlm_RunLengthNonUniformity<br>glrlm_RunLengthNonUniformityNormalized<br>glrlm_RunPercentage<br>glrlm_GrayLevelVariance<br>glrlm_RunVariance<br>glrlm_RunEntropy<br>glrlm_LowGrayLevelRunEmphasis<br>glrlm_HighGrayLevelRunEmphasis<br>glrlm_ShortRunLowGrayLevelEmphasis<br>glrlm_ShortRunHighGrayLevelEmphasis<br>glrlm_LongRunLowGrayLevelEmphasis<br>glrlm_LongRunHighGrayLevelEmphasis
Texture features from NGTDM<br>(n=5)	ngtdm_Coarseness<br>ngtdm_Contrast<br>ngtdm_Busyness<br>ngtdm_Complexity<br>ngtdm_Strength
Texture features from GLDM<br>(n=14)	gldm_SmallDependenceEmphasis<br>gldm_LargeDependenceEmphasis<br>gldm_GrayLevelNonUniformity<br>gldm_DependenceNonUniformity<br>gldm_DependenceNonUniformityNormalized<br>gldm_GrayLevelVariance<br>gldm_DependenceVariance<br>gldm_DependenceEntropy<br>gldm_LowGrayLevelEmphasis<br>gldm_HighGrayLevelEmphasis<br>gldm_SmallDependenceLowGrayLevelEmphasis<br>gldm_SmallDependenceHighGrayLevelEmphasis<br>gldm_LargeDependenceLowGrayLevelEmphasis<br>gldm_LargeDependenceHighGrayLevelEmphasis

Table S2: Parameters used for hyperparameter optimization with GridSearchCV are listed here if these parameters were not chosen from the default option from the scikit-learn package. ElasticNet was used for feature selection. LR: logistic regression, l-SVM: support vector machines (linear kernel), random-forest (RF), MLPC: multi-layer perceptron classifier.

Mode	Parameters
**ElasticNet**	l1_ratio=0.9<br>max_iter=100000<br>alpha= np.logspace(-4, 1, num=10, endpoint=True, base=10.0)
**LR**	C= np.logspace(-6, 3, num=10, endpoint=True, base=10.0)<br>max_iter=1000000<br>solver='lbfgs'
**L-SVM**	C= [0.001, 0.002, 0.005, 0.01, 0.015, 0.02, 0.05, 0.1, 0.15, 0.2, 0.025, 0.5, 1.0, 2, 5, 10, 20, 30, 40, 50, 75, 100]<br>kernel='linear'<br>gamma='auto'<br>max_iter=1000000
**RF**	n_estimators= [10, 25, 50, 75, 100, 125, 150]<br>min_samples_split= [5, 10, 25, 50, 75, 100, 125, 150, 200]<br>max_depth=None
**MLPC**	alpha= np.logspace(-7, 2, num=20, endpoint=True, base=10.0)<br>activation='logistic'<br>max_iter=10000

Table S3: The performance of the machine-learning model using ROC-AUC as a metric was reported here for the training and validation cohorts with a 95% confidence interval (95% CI). CET: contrast-enhancing tumor, NEL: non-enhancing lesion. PeriCET: peritumoral region, WM: white matter. LR: logistic regression, l-SVM: linear support vector machines, RF: random-forest, MLPC: multi-layer perceptron classifier.

**Model**	**ROIs**	**Trainings cohort**		**Validation cohort**	
		**Mean ROC AUC**	**95% CI**	**Mean ROC AUC**	**95% CI**
LR	CET + NEL + PeriCET + WM	0.8641	(0.8204; 0.9079)	0.8147	(0.7372; 0.8922)
	CET	0.9127	(0.8757; 0.9496)	0.8354	(0.7647; 0.9060)
	NEL	0.8076	(0.7548; 0.8604)	0.7644	(0.6765; 0.8521)
	PeriCET	0.8587	(0.8141; 0.9033)	0.8331	(0.7558; 0.9104)
	WM	0.8374	(0.7893; 0.8856)	0.7720	(0.6903; 0.8536)
L-SVM	CET + NEL + PeriCET + WM	0.9372	(0.9085; 0.9659)	0.8280	(0.7517; 0.9043)
	CET	0.9446	(0.9119; 0.9772)	0.7762	(0.6862; 0.8663)
	NEL	0.8630	(0.8159; 0.9101)	0.7491	(0.6477; 0.8504)
	PeriCET	0.8649	(0.8207; 0.9091)	0.8126	(0.7284; 0.8968)
	WM	0.9355	(0.8980; 0.9731)	0.6594	(0.5579; 0.7609)
RF	CET + NEL + PeriCET + WM	1.0	(1.0; 1.0)	0.8173	(0.7356; 0.8990)
	CET	0.9997	(0.9991; 1.0)	0.7904	(0.7078; 0.8730)
	NEL	0.8536	(0.7895; 0.8821)	0.7645	(0.6770; 0.8520)
	PeriCET	0.9585	(0.9387; 0.9782)	0.8079	(0.7234; 0.8924)
	WM	1.0	(1.0; 1.0)	0.7615	(0.6716; 0.8514)
MLPC	CET + NEL + PeriCET + WM	0.9090	(0.8711; 0.9468)	0.8356	(0.7609; 0.9104)
	CET	0.9206	(0.8846; 0.9566)	0.8291	(0.7569; 0.9014)
	NEL	0.8075	(0.7547; 0.8603)	0.7635	(0.6754; 0.8516)
	PeriCET	0.8594	(0.8149; 0.9040)	0.8322	(0.7546; 0.9099)
	WM	0.8745	(0.8304; 0.9185)	0.7519	(0.6680; 0.8359)

Table S4 – S8: A full list of feature importance obtained from every machine-learning model trained with radiomics features from all ROIs (**S4**), contrast-enhancing region (CET, **S5**), non-enhancing lesion (NEL, **S6**), peritumor region (PeriCET, **S7**), and white-matter region (WM, **S8**) are shown in a separate excel-spreadsheet.
